# Intraoperative Surgical Navigation Is as Effective as Conventional Surgery for Zygomaticomaxillary Complex Fracture Reduction

**DOI:** 10.3390/jcm14051589

**Published:** 2025-02-26

**Authors:** Mădălina Bănărescu, Bianca Golzio Navarro Cavalcante, Márton Ács, Bence Szabó, Andrea Harnos, Péter Hegyi, Gábor Varga, Victor Vlad Costan, Gábor Gerber

**Affiliations:** 1 Faculty of Dental Medicine, University of Medicine and Pharmacy “Grigore T. Popa”, 700115 Iasi, Romania; banarescu.madalina@gmail.com (M.B.); victorcostan@gmail.com (V.V.C.); 2 Centre for Translational Medicine, Semmelweis University, 1085 Budapest, Hungary; 3 Department of Oral Biology, Semmelweis University, 1085 Budapest, Hungary; 4 Department of Biostatics, University of Veterinary Medicine Budapest, 1078 Budapest, Hungary; 5 Institute for Translational Medicine, Medical School, University of Pécs, 7624 Pécs, Hungary; 6 Department of Surgery, Oral and Maxillofacial Surgery, Faculty of Dental Medicine, Grigore T. Popa University of Medicine and Pharmacy, 700115 Iasi, Romania; 7 Oral Morphology Group, Department of Anatomy, Histology and Embryology, Semmelweis University, 1085 Budapest, Hungary

**Keywords:** computer-assisted, meta-analysis, midfacial, orbitozygomatic, quadripod, symmetry, tripod

## Abstract

**Background/Objectives**: Zygomaticomaxillary complex (ZMC) fractures are the second most common of all facial bone fractures, and conventional treatment represents a challenge even for experienced surgeons. The aim of this systematic review and meta-analysis was to compare Intraoperative Surgical Navigation (ISN) with conventional surgery in the treatment of ZMC fractures. **Methods**: We reported our systematic review and meta-analysis based on the recommendation of the PRISMA 2020 guideline. The electronic search was conducted on 9 February 2025 in two search engines (PubMed and Web of Science) and two databases (Embase and the Cochrane Library). Randomized controlled trials and observational studies were included. The outcome variables were accuracy, operative time, maximum mouth opening, postoperative stay, amount of bleeding, and cheek numbness. The random-effects model was used for the analysis, and the results were given as mean differences and odds ratios with 95% confidence intervals (CI). After duplicate removal, 1961 articles were found. After the selection procedure, five studies were found to be eligible for qualitative and quantitative analysis. **Results**: There were no statistically significant differences between ISN and conventional surgery regarding the outcomes investigated, except in postoperative average deviation of the zygomatic bone. Our results showed an improvement of 0.64 mm [CI: 0.32, 0.92] zygomatic bone deviation when ISN was used. **Conclusions**: The results suggest that ISN is as effective as the conventional technique in the treatment of ZMC fractures. However, because of the low number of eligible studies, further randomized controlled trials are necessary to strengthen the level of evidence on this matter.

## 1. Introduction

Poorly treated zygomaticomaxillary complex (ZMC) injury is one of the most common post-traumatic problems encountered by craniofacial surgeons today [1]. ZMC fractures account for 25% of all facial bone fractures. Because of its protruded position and convex shape, the ZMC plays a determining role in esthetics, especially when considering the possible postoperative complications, such as malar asymmetry, midfacial widening, and enophthalmos [2]. ZMC injury and postoperative complications can also lead to functional impairment, like diplopia, trismus, and paresthesia [3].

Approximately 80% of displaced ZMC fractures are treated with open reduction and internal fixation [4,5], with rigid fixation using plates and screws forming the accepted standard [1]. The main limitation of this method is that the evaluation of bone repositioning is dependent on visual inspection or palpation. Less experienced surgeons are especially prone to inaccurate evaluation [6], while even experienced surgeons are likely to consider the treatment challenging, such that 10–15% of patients are left with some degree of midfacial deformity after conventional surgery. Accordingly, the main cause of reoperation is patient dissatisfaction with appearance [7].

Currently, computer-based surgery is rapidly emerging in the surgical field [8]. Intraoperative Surgical Navigation uses technology similar to the global positioning systems used in automobiles. It has three primary components: a localizer, an instrument or surgical probe, and a CT scan data set [9]. It allows the surgeon to determine the accurate 3D location of fracture segments, surgical instruments, and surrounding anatomical structures [10]. Intraoperative Surgical Navigation (ISN) systems were first used in neurosurgery [11] before being applied in a variety of oral and maxillofacial surgeries [12,13]. The research suggests that navigation systems can accurately guide the operator during interventions and can reduce the operative time [14,15,16,17,18].

However, it remains unclear whether ISN is superior to conventional surgery when treating ZMC fractures in terms of postoperative clinical outcomes. Recent studies suggest that ISN improves fracture reduction accuracy and orbital volume reconstruction without an augmented risk of complications [19,20]. Even so, a comprehensive analysis is still needed. A recent meta-analysis approached this issue but provided only a very low level of evidence [20].

Therefore, we conducted a meta-analysis with the aim of investigating whether ISN can achieve better clinical outcomes than conventional surgery in the treatment of ZMC fractures.

## 2. Materials and Methods

We reported our systematic review and meta-analysis based on the recommendation of the PRISMA (Preferred Reporting Items for Systematic Reviews and Meta-Analyses) 2020 guideline [21] (Table S2), and we followed the Cochrane Handbook [22] (PROSPERO—International Prospective Register of Systematic Reviews registration number CRD42022380656, registered on 13 December 2022).

### 2.1. Information Sources and Search Strategy

Our systematic search was conducted on 9 February 2025 in two search engines (PubMed and Web of Science) and two databases (Embase and the Cochrane Library). No filters and restrictions were applied. The search key can be found in Table S1. An additional manual search based on the reference list of included articles was performed.

### 2.2. Selection Process

Duplicates were removed by title, abstract, and full text, after which the selection was performed by two independent review authors (MB and MA). Any disagreements were resolved by a third independent reviewer (BGNC).

### 2.3. Eligibility Criteria

The population–intervention–control–outcome (PICO) framework was used to formulate the research questions. Studies reporting on patients with zygomaticomaxillary complex fractures (P) undergoing conventional surgery assisted by Intraoperative Surgical Navigation (I) and conventional surgery (C) were included in the analysis. ISN was defined as real-time 3D navigation, while conventional surgery consisted of open reduction and internal fixation. Primary outcomes (O) were zygomatic eminence accuracy, infraorbital rim accuracy, and average deviation of the zygomatic bone. Zygomatic eminence accuracy was defined as the 2D linear deviation of the most prominent point of the zygomatic bone measured from the midsagittal plane. Infraorbital rim accuracy was defined as 2D linear deviation of the articulation of the zygomatic bone at the level of inferior orbital rim, measured from the midsagittal plane. Average deviation (3D) of the zygomatic bone’s surface was measured by superimposing the pre-operative virtual planning with the postoperative imaging. The secondary outcomes (O) were operative time, maximum mouth opening, amount of bleeding, postoperative stay, orbital volume, diplopia, enophthalmos, and complications, such as cheek numbness, wound infection, and screw loosening.

Regarding inclusion criteria for study type, full-text publications reporting primary data, such as RCT (randomized controlled trial), non-RCTs, and observational studies, were included. Outcomes were included if at least two publications reporting on them could be found.

Regarding exclusion criteria, reviews, case series, case reports, and descriptive studies were excluded. Also excluded were studies reporting on patients under 16 years old, patients with soft-tissue defects or insufficient tissue to cover the postoperative wound, and CT- and C-arm-based surgical navigation.

### 2.4. Data Collection Process

Two authors (MB, MA) independently collected the following data from the eligible articles: first author, year of publication, country, number of centers, study period and design, demographic data, brand of navigation system, and outcome-related data. A third independent reviewer (BGNC) resolved any disagreements. A standardized data collection form in an Excel spreadsheet was used. In the case of any missing data or uncertain outcome definition, authors of the given article were contacted.

### 2.5. Study Risk of Bias Assessment

The risk of bias assessment was performed based on the recommendation of the Cochrane Collaboration [23] by two authors independently (MB, MA) using RoB2 for randomized control trials and ROBINS-I for non-randomized studies. RoB2 is structured into five domains of bias, each with a series of signaling questions, indicating ‘Low’ or High’ risk of bias or ‘Some concerns’ [24]. The ROBINS-I tool covers seven domains: the first two domains address issues before intervention, the third addresses the classification of the interventions, and the remaining four address issues arising after the start of the intervention. The judgment can be ‘Low’, ‘Moderate’, ‘Serious’, or ‘Critical’ risk of bias; alternatively, the judgment ‘No information’ is applied in the case of insufficient data [25]. A third independent reviewer (BGNC) resolved any disagreement.

### 2.6. Certainty of Evidence

GRADEproGDT (Guideline Development Tool, https://www.gradepro.org/) was used, and each outcome was tested for the factors that can reduce the quality of evidence, such as study design, risk of bias, inconsistency of results, indirectness of evidence, imprecision, and publication bias. Certainty of evidence was downgraded by one or two levels for serious and very serious concerns, respectively. The GRADE approach can result in one of four grades: ‘High’, ‘Moderate’, ‘Low’, or ‘Very Low’ [26]. The assessment was performed independently by two authors (MB, MA), while a third (BGNC) resolved any disagreement.

### 2.7. Synthesis Methods

Prior to analysis, we assumed considerable between-study heterogeneity; accordingly, random-effect models were used to pool effect sizes.

For continuous outcomes, the mean difference (MD) was used for the effect size measure, with 95% confidence interval (CI). The study MDs and pooled MDs were calculated by extracting the sample size, the mean, and the corresponding standard deviation (SD) from each study (in each group separately). Results were reported as experimental group values minus control group values. Inverse variance weighting method was used to calculate the pooled MD. If the quartiles were given instead of the mean and SD, then the Lou and Shi methods were used for calculating the mean and standard deviation [27,28] as implemented in the meta R package (https://www.r-project.org/). As a limitation, it should be highlighted that this is an estimation. With regard to maximal mouth opening, only the postoperative values were reported in two articles [29,30], so for the analysis, the postoperative mouth opening value was used. Zhu Cheng [7] reported only the baseline and the change from baseline mean and SD-s for the maximal mouth opening. Given these two data pairs, the postoperative mean value for this article could be calculated by adding the baseline and the change from baseline mean values. However, based on only these data, the postoperative SD value for the Zhu Cheng [7] article could not be determined precisely. Consequently, the postoperative SD was calculated using different correlation coefficients (−1; −0.5; 0; 0.5; 1) following the recommendations of the *Cochrane Handbook* [22]. The results utilizing the Zhu Cheng [7] article with 0 correlation were reported in the main text, while the others were reported in a separate table and supplementary figures.

An odds ratio (OR) with a 95% confidence interval (CI) was used to measure the effect size of binary data. To calculate this study’s odds ratios and the pooled odds ratio, the total number of patients and those with the event of interest in each group separately (referred to as “raw data”) was extracted or calculated from the studies where it was available. The results were reported as the odds of event of interest in experimental group versus the odds of event of interest in the control group. In the case that only OR without “raw data” was given, the OR and its 95% confidence interval were used (assuming Wald-type interval if not given).

A Hartung–Knapp adjustment was used for CIs. The HK adjustment was used because the number of studies was low. The random-effects meta-analysis uses a normal approximation to estimate the confidence interval of the overall effect, which can lead to overly narrow CIs, especially in the case of a low number of studies. The HK adjustment aims to provide us with more robust—albeit wider—confidence intervals by some adjustments in the estimation process [31,32]. To estimate the heterogeneity variance measure (τ 2) for MD, the restricted maximum likelihood estimator was used with the Q profile method for confidence interval [33,34].

Additionally, between-study heterogeneity was described by Higgins and Thompson’s I^2^ statistics [35].

Results were considered statistically significant if the pooled CI did not contain the null value. The findings related to meta-analysis were summarized using forest plots. Due to the low number of studies, prediction intervals (i.e., the expected range of effects of future studies) of results were not reported.

As the total number of studies included in the meta-analysis was very low, publication bias could not be assessed. Egger’s tests were not performed, as they lack power below at least 10 studies [36,37].

All statistical analyses were made with R [38] using the meta [39] package for basic meta-analysis calculations and plots.

## 3. Results

### 3.1. Search and Selection

**Figure 1:**
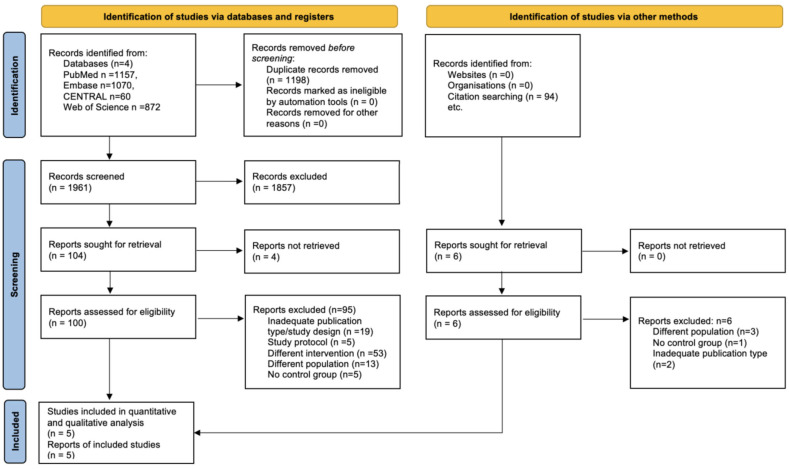
PRISMA 2020 flowchart representing the study selection process.

Our search identified 1841 studies, of which 1336 remained after duplicate removal. Following the title and abstract search, 35 studies were eligible for full-text selection. Finally, five studies were included in the meta-analysis. The main reasons for exclusion were inadequate publication type or study design, different interventions or populations, and lack of a control group (Figure 1).

### 3.2. Study Characteristics

#### 3.2.1. Description of Excluded Studies

After full-text analysis, ninety-nine studies were excluded. Nineteen were reviews, case reports, or correspondence; five were study protocols; fifty-two used intraoperative CT, 3D C-arm navigation, ultrasonography, or endoscopy; four did not investigate ZMC fractures; five lacked a control group; thirteen included pediatric patients or included zygomatic arch fractures; and one did not investigate ISN. CT and 3D C-arm imaging studies were excluded because they are not real-time navigation systems. Finally, only five articles were eligible for inclusion in the quantitative and qualitative analysis.

#### 3.2.2. Description of Included Studies

All studies were published between 2012 and 2021 (Table 1). Three RCTs [7,29,40] and two retrospective studies [30,41] reporting on 189 patients with unilateral zygomatic fractures were included in the analysis: 97 in the navigation group (29.7% female) and 92 in the control group (33.1% female). The patients had suffered type B and type C zygomatic fractures (Zingg classification [4]), both acute and delayed. The follow-up time ranged from 6 to 18 months.

#### 3.2.3. Risk of Bias Assessment

We detected a critical risk in Lee Yang [30] and Yu Bao [41] because of bias due to the selection of participants, which was based on the characteristics observed after the start of the intervention. Additionally, the start of the follow-up and intervention did not coincide for most of the participants. The studies of He Gong [40] and Zhu Cheng [7] provided no information about the allocation sequence being concealed until participants were enrolled and assigned to the interventions. None of the included RCTs [7,29,40] had a complete pre-specified analysis plan, including all the outcomes reported. For these reasons, there were some concerns in the risk-of-bias assessment (Figures S1 and S2).

#### 3.2.4. Certainty of Evidence

The assessment of certainty in the body of evidence was ‘Very low’ (Figure S3). The domain of limitations in study design or execution was downgraded by one level due to the included non-randomized studies in our analysis and the high risk of bias. Inconsistency downgraded this study by one level due to the substantial degree of heterogeneity. Imprecision was downgraded because of the low sample size. There was no reason to suspect the presence of publication bias.

### 3.3. Primary Outcome (Accuracy)

#### 3.3.1. Zygomatic Eminence Accuracy

**Figure 2:**
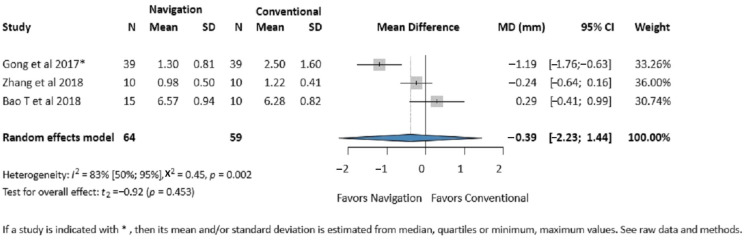
Forest plot for the zygomatic eminence accuracy comparing the Intraoperative Surgical Navigation and the conventional surgery groups. MD, mean difference. CI, confidence interval. mm, millimeters [29,40,41].

Three studies (123 patients) reported on the zygomatic eminence accuracy [29,40,41]. No significant difference was found between the two groups (MD −0.39 mm, 95% CI: −2.23, 1.44; *I*^2^ = 83%, *p*-value 0.453) (Figure 2).

#### 3.3.2. Infraorbital Rim Accuracy

**Figure 3:**
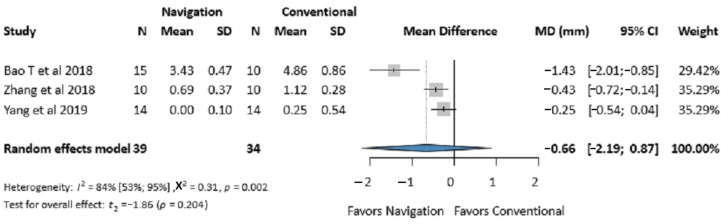
Forest plot for the infraorbital rim accuracy comparing the Intraoperative Surgical Navigation and the conventional surgery groups. MD, mean difference. CI, confidence interval. mm, millimeters [29,30,40].

Three studies (73 patients) reported on infraorbital rim accuracy [29,30,41]. No significant difference was found between the two groups (MD −0.66 mm, 95% CI: −2.19, 0.87; *I*^2^ = 84%, *p*-value 0.204) (Figure 3).

#### 3.3.3. Average Deviation of the Zygomatic Bone

**Figure 4:**
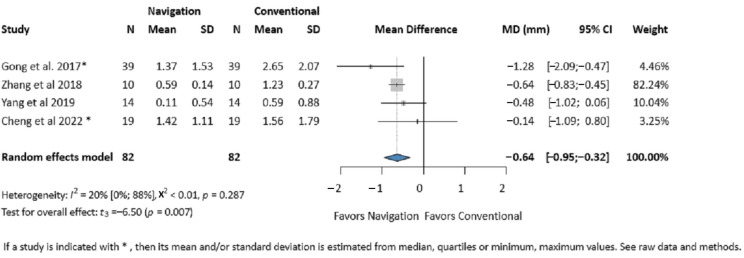
Forest plot for postoperative average deviation of the zygomatic bone comparing the Intraoperative Surgical Navigation and the conventional surgery groups. MD, mean difference. CI, confidence interval. mm, millimeters [7,29,30,40].

Four studies (164 patients) reported on the average deviation [7,29,30,40]. Our results showed significant improvement in the navigation group compared to the control group (MD 0.64 mm, 95% CI: 0.32, 0.95, *I*^2^ = 20%, *p*-value 0.007). A tendency towards better accuracy in more severe and delayed fractures can also be observed (Figure 4).

### 3.4. Secondary Outcomes

#### 3.4.1. Operative Time

**Figure 5:**
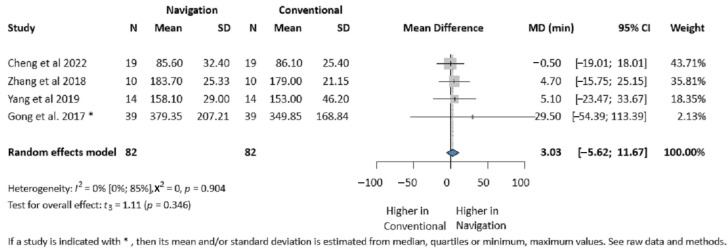
Forest plot for the operative time comparing the Intraoperative Surgical Navigation and the conventional surgery groups. MD, mean difference. CI, confidence interval. min, minutes [7,29,30,40].

Four studies (164 patients) reported on the operative time [7,29,30,40]. No significant difference was found between the two groups (MD 3.03 min, 95% CI: −5.62, 11.67; *I*^2^ = 0%, *p*-value 0.346) (Figure 5).

#### 3.4.2. Mouth Opening

**Figure 6:**
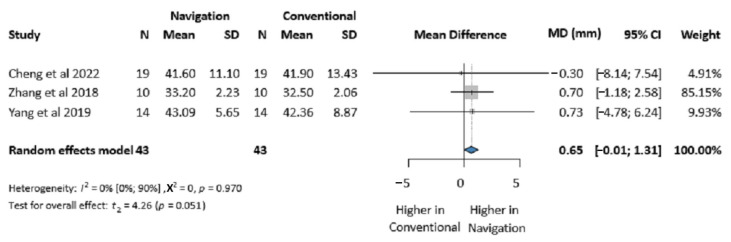
Forest plot for the postoperative maximum mouth opening comparing the Intraoperative Surgical Navigation and the conventional surgery groups. MD, mean difference. CI, confidence interval. mm, millimeters [7,29,30].

Three studies (86 patients) reported maximum mouth opening [7,29,30]. For measurement pairs carried out on the same patients before and after a treatment, negative correlations are unlikely. We reported the calculations based on 0 correlation as a conservative approach. For the calculation based on 0 correlation between the baseline and the follow-up measurements, no significant difference was found between the two groups (MD 0.65 mm, 95% CI: −0.01, 1.31; *I*^2^ = 0%, *p*-value 0.970) (Figure 6). The different correlation coefficient-based calculations are presented in Table 2, and the forest plots are presented in the Supplementary Materials (Figures S4–S7).

#### 3.4.3. Postoperative Stay and Amount of Bleeding

These outcomes were each reported by only two studies; therefore, no relevant conclusions can be drawn. Based on these two studies, there was no significant difference between the two groups regarding postoperative stay (MD 0.19 days, 95% CI: −5.31, 4.94) [30,40] or for the amount of bleeding (MD 14.85 mL, 95% CI: −53.92, 83.63) [7,40] (Figures S8 and S9).

#### 3.4.4. Complications (Cheek Numbness, Screw Loosening, Wound Infection)

Two studies (106 patients) reported on cheek numbness [30,40]. Based on these two studies, no significant difference was found between the two groups (OR 0.91, 95% CI: 0.00, 2121.97) (Figure S10). No other postoperative complications were reported.

#### 3.4.5. Orbital Volume, Diplopia, Enophthalmos

Since only one study per outcome was found, it was not possible to perform any analysis for these outcomes. Ye Zhang [29] reported on one patient from the control group with a partially resolved diplopia. He Gong [40] reported on enophthalmos in 10 vs. 8 patients in the navigation and control group, respectively. Only Yu Bao [41] investigated the reduced orbital volume, reporting 2.15 ± 1.4 cm^3^ in the navigation group and 1.6 ± 0.64 cm^3^ in the control group (*p*-value < 0.05).

#### 3.4.6. Publication Bias and Heterogeneity

When a meta-analysis includes less than 10 studies, the power of the test for funnel plot asymmetry is insufficient to draw a distinction between real asymmetry and simple chance [42]. Therefore, we could not perform this test.

Except for the average deviation, operative time, and mouth opening, the *p* values related to I^2^ indicated a substantial degree of heterogeneity. The main reason for this was the different types of fractures investigated. The test for heterogeneity for outcomes reported by only two studies could not be performed.

## 4. Discussion

Proper reduction and fixation of displaced zygomatic fractures are essential to ensure proper healing and prevent postoperative complications [43]. Five studies with 189 patients were included to compare the clinical outcomes of ISN and conventional surgery for treating ZMC fractures. The present study investigated the accuracy, operative time, mouth opening, postoperative stay, amount of bleeding, and cheek numbness. The results for most of the outcomes did not differ significantly between the two methods; however, the average deviation of the zygomatic bone was significantly improved by the use of ISN. Hence, our data show no disadvantage in postoperative outcomes for navigation-assisted surgery compared to the conventional technique.

A recent meta-analysis [20] suggested that there is a significant improvement in the accuracy reduction, favoring ISN. However, that meta-analysis included both 2D and 3D measurements in the same analysis, which is highly questionable. Moreover, in performing subgroup analysis, no difference was shown in the 2D group [20]. The present study provides a more comprehensive analysis, including the zygomatic eminence and infraorbital rim accuracy, as well as an analysis of the 3D average deviation. Investigating the accuracy of different landmarks yielded additional information. The present study also investigated other outcomes, such as mouth opening, length of hospital stay, and cheek numbness.

Zygomatic bone forms the most anterolateral projection on each side of the middle face [44]. Facial symmetry is achieved by restoring the three-dimensional position of the malar prominence [1]. Studies investigating the accuracy of the conventional technique are divided. Tavosi Khaqani [45] showed that the conventional technique could not accurately reduce ZMC fractures. Others found that even when similar accuracy was achieved compared to a healthy zygoma, it was not possible to restore the antero-posterior dimension of the zygomatic eminence [46,47]. When ISN is applied, the accuracy ranges from 0.46 to 1.22 mm [48,49]. We investigated the 2D difference from the midsagittal plane between the affected and unaffected sides. Our results showed an improvement of 0.39 mm in favor of the navigation group but with no significant difference. The values reported by Zhu Cheng [7] could not be included in our analysis, as they did not report the standard deviations; however, they found similar results to ours, although they only investigated acute type B fractures [4].

The infraorbital rim is an important site for fracture reduction and is often displaced by an ipsilateral naso-orbito-ethmoid fracture. The position of the globe is affected by the direction and degree of displacement of the zygoma. Achieving the correct orbital volume in complex fractures is even more challenging and is more commonly associated with the need for secondary procedures [1]. The same authors, in two separate studies, provided postoperative measurements (mm) for infraorbital rim (mean 0.07, range 0–0.55; 0.07 ± 0.26, range: 0.00–1.14) and also for orbital volume (mm^3^): mean 1.05, range 0.12–3.61 [49,50]. Shi Zong [51] showed a significant improvement in orbital volume when using ISN (0.57 ± 0.43 vs. 1.60 ± 0.78). Li He [52] investigated globe projection and the necessity of ocular prosthesis, with their results favoring ISN. Our present data show an improvement of 0.66 mm in infraorbital rim accuracy in favor of ISN, but no significant difference was found. The reason for this could be the low number of studies included in our analysis.

Reduction and fixation of three of the four potential points of fixation (the lateral orbital rim, the inferior orbital rim, the zygomaticomaxillary buttress, and the zygomatic arch) will correct both translation and rotation of the zygoma in three-dimensional space [1]. Due to the heterogeneity of the accuracy measurements between studies in the literature, it was not possible to investigate all four fixation points. Therefore, analysis was conducted on the postoperative average deviation of the zygomatic bone. ISN significantly improves postoperative average deviation. This is also supported by other findings in the literature [52], while postoperative average deviation ranged from 1.24 to 1.57 [48,53]. In healthy individuals, the zygoma asymmetry is between 0.8 and 1.6 mm [7], which suggests ISN could accurately guide the 3D reduction of ZMC fractures.

Bouchard Bergeron [17] showed a 36.1% surgical time reduction when using the navigation system. By contrast, our analysis showed that using a navigation system does not improve the operative time. Meanwhile, an extra 30–40 min was needed to install the digital reference frame, register images of the patient, and recalibrate anatomic landmarks [29,40]. It should be noted that the surgeons’ experience with the system was unknown. Shi Zong [51] reported that navigation reduced the operative time compared to the conventional group but without a significant difference. The learning curve is an important factor, as the same intervention initially takes longer with the navigation system [54]. Accordingly, Duclos Maruthappu [55] showed how the operative time constantly decreases when surgical experience increases, with some interventions being reduced to half the time. This observation is also supported by Jamison Hopper [56].

The maxillo-facial region has a high degree of anatomical complexity, with an abundance of nerves and vessels. Moreover, the zygoma is the key to restoring facial and orbital projection in severely displaced and comminuted fractures [57]. Therefore, three-dimensional imaging is highly important. Our present results are promising, and we can also state that navigation systems have some advantages. They can reduce the incidence of repeated procedures, they can facilitate surgery when dealing with soft-tissue lesions where access is limited by allowing for minimally invasive access, and they can demonstrate proper reduction without the need to obtain a postoperative CT scan and without exposing the patient to additional radiation [18]. These aspects are even more important in severe displacement cases of fractured bone or comminuted fractures, for which the reduction is more difficult [58]. Also, secondary correction of deformities related to the untreated or poorly treated displaced zygoma is challenging and often limited in success because of bony malunion and soft-tissue contracture [1]. Koulechov Strauss [59] showed that ISN provides additional relevant information, with 47.9% of ISN applications leading to a change in surgical strategy. Moreover, these changes were more common in the hands of less experienced surgeons. The use of a navigation system in mid-facial reconstruction is still an emerging technology, which makes the learning curve highly important. The learning curve points to a key role in training surgeons to facilitate improved performance and accuracy and reduce surgical time [56]. Hence, the full benefits of navigation-assisted surgery will only be realized once surgeons grow more skilled in this technique.

## 5. Strengths

To the best of our knowledge, this study is the most comprehensive analysis to date of the accuracy of the intervention, which was measured at different landmarks (zygomatic eminence, infraorbital rim) and with respect to the 3D average deviation of the zygomatic bone. Also included are a series of other outcomes related to functionality (mouth opening, cheek numbness) and surgical-related events (operative time, amount of bleeding). This study benefits from a rigorous methodology according to the present guidelines, and the protocol was registered in advance.

## 6. Limitations

The major limitation of this study was related to the different types of fractures that were investigated: type B and C [4], both acute and delayed. Secondly, none of the included studies investigated soft tissue, to which equal importance should properly be attributed. Careful exposure of the bony surfaces and resuspension of the soft tissues after repositioning and fixation of the fractures is crucial for long-term treatment success [60]. Even with accurate reduction of the bone anatomy, soft-tissue deformities can result from inadequate suspension after extensive surgical exposure [1]. Some authors even suggest that midface soft-tissue resuspension should be performed following ZMC reduction and fixation to prevent unwanted soft tissue descent and lower lid malposition [61].

The zygomaticosphenoid alignment at the lateral orbital wall is recognized as a fundamental key to the proper reduction of orbitozygomatic injuries. It also greatly impacts the orbital volume [1]. Lee Yang [30] found a significant difference in accuracy at the zygomaticosphenoid alignment in favor of navigation. Since only one study investigated this landmark, we could not include it in our analysis; however, given its importance, further studies should prioritize this.

One study [40] reported the values as medians; consequently, the mean values and standard deviations had to be estimated. For another study [7], a formula provided by the authors was used to calculate the linear deviation from translational error.

A low number of studies were included overall, which downgraded the level of evidence due to the low sample size and limited the generalizability of our findings. Therefore, observational studies were also included in the analysis, and a degree of bias due to the selection of participants could not be avoided.

For these reasons, and with respect to some concerns over the high risk of bias in some of the domains and with the very low level of evidence, the results should be carefully interpreted.

### 6.1. Implication for Practice

Our results indicate that navigation-assisted surgery can achieve the accurate reduction of ZMC fractures. Navigation-assisted surgery could also offer some advantages when treating delayed fractures or when assisting secondary reconstruction. Moreover, this system could be beneficial for inexperienced surgeons, for whom accurate assessment of the reduction is especially challenging.

### 6.2. Implication for Research

Based on our results, further randomized controlled studies are needed to evaluate the efficiency of Intraoperative Surgical Navigation in treating zygomatic fractures. Patients suffering from type B and type C fractures, as well as acute and delayed cases, should be investigated separately. The time spent using the navigation system during the surgery should be recorded, as well as the experience of the surgeons with the system. Regarding mouth opening, pre-operative measurements might give additional insight. Moreover, studies reporting on the accuracy concerning the zygomaticosphenoid alignment, soft-tissue management, and cost-efficiency are needed.

## 7. Conclusions

In conclusion, the present results suggest that ISN and the conventional technique are equally effective in the treatment of ZMC fractures. The postoperative average deviation of the zygomatic bone is improved by ISN, and there were no significant differences between the two methods for the other outcomes. The data show navigation-assisted surgery to be more accurate in type C and delayed fractures. Our results indicate that further studies are needed to determine if the advantages outweigh the additional cost of the system.

## Figures and Tables

**Table 1:** Basic characteristics of included studies.

Author, Year	Zhang et al., 2018 [29]	Cheng et al., 2022 [7]	Bao T et al., 2018 [41]	Yang et al., 2019 [30]	Gong et al., 2017 [40]
Country (center)	China (single-center)	China (single-center)	China (single-center)	Taiwan (single-center)	China (single-center)
Type of study	RCT	RCT	Retrospective	Retrospective	RCT
Sample size (navigation)	10	19	15	14	39
Sample size (conventional)	10	19	10	14	39
Type of fracture	Unilateral ZMC fractures of type B with delayed surgery and/or bone defect and type C	Unilateral type B ZMC fractures	Acute unilateral type C fractures	Acute unilateral type B and simple type C fractures	Delayed unilateral ZMC fractures of type B and type C
Navigation system	VectorVision 2 navigation system (BrainLAB)	Acc-Navi system (Multi- functional Surgical Navigation System, Shanghai, China)	NR	Kolibri workstation Platform 2.0 (BrainLAB, Feldkirchen, Germany)	VectorVision navigation system (BrainLAB)
Examined outcome	Zygomatic eminence accuracy, IO accuracy, average deviation, operative time, maximum mouth opening	Average deviation, operative time, maximum mouth opening, amount of bleeding	Zygomatic eminence accuracy, IO accuracy	IO accuracy, average deviation, operative time, maximum mouth opening, cheek numbness, postoperative stay	Zygomatic eminence accuracy, average deviation, operative time, cheek numbness, postoperative stay, amount of bleeding

RCT randomized controlled trial; ZMC zygomaticomaxillary complex; NR not retrieved.

**Table 2:** Summarized results of the meta-analyses of maximum mouth opening mean differences based on different correlation coefficients used for the calculation of Zhu Cheng [7] follow-up SD-s. cc. stands for correlation coefficient, MD stands for mean difference, and * marks a significant difference from 0.

Cheng, Zhu [7]	MD	MD—95% CI	*I*^2^ & 95% CI	*p*-Value
−0.1	0.68	[0.20; 1.16] *	0% [0–90%]	0.985
−0.5	0.67	[0.16; 1.19] *	0% [0–90%]	0.982
0	0.65	[−0.01; 1.31]	0% [0–90%]	0.970
0.5	0.56	[−0.52; 1.64]	0% [0–90%]	0.915
1	−0.25	[−0.90; 0.40]	0% [0–90%]	0.544

## Data Availability

The raw data supporting the conclusions of this article will be made available by the authors upon request.

## Supplementary Materials

The following supporting information can be downloaded at https://www.mdpi.com/article/10.3390/jcm14051589/s1. Figure S1: Risk of bias assessment for each outcome for RCTs (ROB2 tool); Figure S2: Risk of bias assessment for each outcome for retrospective studies (ROBINS-I tool); Figure S3: Certainty of evidence for each outcome (GRADE table); Figure S4: Forest plot for the postoperative maximum mouth opening comparing the Intraoperative Surgical Navigation and the conventional surgery groups (0.5 correlation). MD, mean difference. CI, confidence interval. mm, millimeters; Figure S5: Forest plot for the postoperative maximum mouth opening comparing the Intraoperative Surgical Navigation and the conventional surgery groups (1 correlation). MD, mean difference. CI, confidence interval. mm, millimeters; Figure S6: Forest plot for the postoperative maximum mouth opening comparing the Intraoperative Surgical Navigation and the conventional surgery groups (−1 correlation). MD, mean difference. CI, confidence interval. mm, millimeters; Figure S7: Forest plot for the postoperative maximum mouth opening comparing the Intraoperative Surgical Navigation and the conventional surgery groups (−0.5 correlation). MD, mean difference. CI, confidence interval. mm, millimeters; Figure S8: Forest plot for the postoperative stay comparing the Intraoperative Surgical Navigation and the conventional surgery groups. MD, mean difference (days). CI, confidence interval; Figure S9: Forest plot for the amount of bleeding comparing the Intraoperative Surgical Navigation and the conventional surgery groups. MD, mean difference (milliliters). CI, confidence interval; Figure S10: Forest plot for the cheek numbness comparing the Intraoperative Surgical Navigation and the conventional surgery groups. OR, odds ratio. CI, confidence interval; Table S1: Search key; Table S2: Prisma checklist.
