# Supplementary material for: Intraoperative Surgical Navigation Is as Effective as Conventional Surgery for Zygomaticomaxillary Complex Fracture Reduction
Source: J Clin Med. 2025 Feb 26;14(5):1589. doi: 10.3390/jcm14051589 (PMC11899977; doi:10.3390/jcm14051589)
Supplement: Supplementary file 1 [file jcm-14-01589-s001.zip › jcm-3454810-supplementary.pdf]

Table S1. Search key

**PubMed**	**((‘Zygomatic Fractures’[Mesh] OR ‘Zygoma’[Mesh] OR ‘zygoma’ OR ‘zygomatic’ OR ‘orbitozygomatic’ OR ‘orbital zygoma’ OR malar OR trimalar OR tripod* OR quadripod*) AND ("Fractures, Bone"[Mesh:NoExp] OR fracture)) AND (‘computer-assisted’ OR ‘computer-aided’ OR ‘navigation’ OR management OR preoperative-plan* OR planning OR ‘pre operative plan’ OR pre-surg* OR pre plan* OR preplan* OR preoperative-care OR preparation* OR surgical plan OR 'virtual surgical plan’ OR ‘intraoperative surgical navigation’)**
**Cochrane**	**((‘zygoma’ OR ‘zygomatic’ OR ‘orbitozygomatic’ OR ‘orbital zygoma’ OR malar OR trimalar OR tripod* OR quadripod*) AND (fracture)) AND (‘computer-assisted’ OR ‘computer-aided’ OR ‘navigation’ OR management OR preoperative-plan* OR planning OR pre-operative-plan* OR pre-surg* OR preplan* OR preoperative-care OR preparation* OR surgical plan OR 'virtual surgical plan’ OR ‘intraoperative surgical navigation’)**
**Embase**	**('zygoma arch fracture'/exp OR 'zygoma arch fracture*':ti,ab,kw OR 'tripod*':ti,ab,kw OR 'quadripod*':ti,ab,kw OR ('zygoma'/exp AND 'fracture'/de) OR 'zygoma*' OR 'orbitozygomatic' OR 'malar' OR 'trimalar') AND 'fracture*':ti,ab,kw AND ('three-dimensional imaging'/exp OR 'three dimensional':ti,ab,kw OR '3d':ti,ab,kw OR '3 d':ti,ab,kw OR 'virtual reality'/exp OR 'virtual*':ti,ab,kw OR 'reconstructive surgery'/exp OR 'reconstructive surg*':ti,ab,kw OR 'computer assisted':ti,ab,kw OR 'computer aided':ti,ab,kw OR navigation*:ti,ab,kw OR management:ti,ab,kw OR 'preoperative plan*':ti,ab,kw OR 'planning*':ti,ab,kw OR 'pre operative plan*':ti,ab,kw OR 'presurg*':ti,ab,kw OR 'pre plan*':ti,ab,kw OR 'preplan*':ti,ab,kw OR 'preoperative care':ti,ab,kw OR 'preparation*':ti,ab,kw)**
**Web of Science**	**(((‘zygomat*’ OR ‘orbitozygomatic’ OR ‘orbital zygoma’ OR malar OR trimalar OR tripod* OR quadripod*) AND (fracture)) AND (‘computer-assisted’ OR ‘computer-aided’ OR ‘navigation’ OR management OR preoperative-plan* OR planning OR pre-operative-plan* OR pre-surg* OR preplan* OR preoperative-care OR preparation* OR surgical plan OR 'virtual surgical plan’ OR ‘intraoperative surgical navigation’))**

Table S2. Prisma Checklist

Section and Topic	Item #	Checklist item	Location where item is reported
**TITLE**			
Title	1	Identify the report as a systematic review.	1
**ABSTRACT**			
Abstract	2	See the PRISMA 2020 for Abstracts checklist.	1
**INTRODUCTION**			
Rationale	3	Describe the rationale for the review in the context of existing knowledge.	1,2
Objectives	4	Provide an explicit statement of the objective(s) or question(s) the review addresses.	2
**METHODS**			
Eligibility criteria	5	Specify the inclusion and exclusion criteria for the review and how studies were grouped for the syntheses.	2,3
Information sources	6	Specify all databases, registers, websites, organisations, reference lists and other sources searched or consulted to identify studies. Specify the date when each source was last searched or consulted.	2
Search strategy	7	Present the full search strategies for all databases, registers and websites, including any filters and limits used.	2
Selection process	8	Specify the methods used to decide whether a study met the inclusion criteria of the review, including how many reviewers screened each record and each report retrieved, whether they worked independently, and if applicable, details of automation tools used in the process.	2,3
Data collection process	9	Specify the methods used to collect data from reports, including how many reviewers collected data from each report, whether they worked independently, any processes for obtaining or confirming data from study investigators, and if applicable, details of automation tools used in the process.	2,3
Data items	10a	List and define all outcomes for which data were sought. Specify whether all results that were compatible with each outcome domain in each study were sought (e.g. for all measures, time points, analyses), and if not, the methods used to decide which results to collect.	2,3
	10b	List and define all other variables for which data were sought (e.g. participant and intervention characteristics, funding sources). Describe any assumptions made about any missing or unclear information.	2,3
Study risk of bias assessment	11	Specify the methods used to assess risk of bias in the included studies, including details of the tool(s) used, how many reviewers assessed each study and whether they worked independently, and if applicable, details of automation tools used in the process.	3
Effect measures	12	Specify for each outcome the effect measure(s) (e.g. risk ratio, mean difference) used in the synthesis or presentation of results.	3,4
Synthesis methods	13a	Describe the processes used to decide which studies were eligible for each synthesis (e.g. tabulating the study intervention characteristics and comparing against the planned groups for each synthesis (item #5)).	3,4
	13b	Describe any methods required to prepare the data for presentation or synthesis, such as handling of missing summary statistics, or data conversions.	3,4
	13c	Describe any methods used to tabulate or visually display results of individual studies and syntheses.	3,4
	13d	Describe any methods used to synthesize results and provide a rationale for the choice(s). If meta-analysis was performed, describe the model(s), method(s) to identify the presence and extent of statistical heterogeneity, and software package(s) used.	3,4
	13e	Describe any methods used to explore possible causes of heterogeneity among study results (e.g. subgroup analysis, meta-regression).	3,4
	13f	Describe any sensitivity analyses conducted to assess robustness of the synthesized results.	3,4
Reporting bias assessment	14	Describe any methods used to assess risk of bias due to missing results in a synthesis (arising from reporting biases).	3
Certainty assessment	15	Describe any methods used to assess certainty (or confidence) in the body of evidence for an outcome.	3
**RESULTS**			

Section and Topic	Item #	Checklist item	Location where item is reported
Study selection	16a	Describe the results of the search and selection process, from the number of records identified in the search to the number of studies included in the review, ideally using a flow diagram.	4
	16b	Cite studies that might appear to meet the inclusion criteria, but which were excluded, and explain why they were excluded.	4
Study characteristics	17	Cite each included study and present its characteristics.	5
Risk of bias in studies	18	Present assessments of risk of bias for each included study.	6
Results of individual studies	19	For all outcomes, present, for each study: (a) summary statistics for each group (where appropriate) and (b) an effect estimate and its precision (e.g. confidence/credible interval), ideally using structured tables or plots.	6,7
Results of syntheses	20a	For each synthesis, briefly summarise the characteristics and risk of bias among contributing studies.	6
	20b	Present results of all statistical syntheses conducted. If meta-analysis was done, present for each the summary estimate and its precision (e.g. confidence/credible interval) and measures of statistical heterogeneity. If comparing groups, describe the direction of the effect.	6,7,8,9
	20c	Present results of all investigations of possible causes of heterogeneity among study results.	11
	20d	Present results of all sensitivity analyses conducted to assess the robustness of the synthesized results.	8
Reporting biases	21	Present assessments of risk of bias due to missing results (arising from reporting biases) for each synthesis assessed.	-
Certainty of evidence	22	Present assessments of certainty (or confidence) in the body of evidence for each outcome assessed.	6
**DISCUSSION**			
Discussion	23a	Provide a general interpretation of the results in the context of other evidence.	9,10
	23b	Discuss any limitations of the evidence included in the review.	11
	23c	Discuss any limitations of the review processes used.	11
	23d	Discuss implications of the results for practice, policy, and future research.	11,12
**OTHER INFORMATION**			
Registration and protocol	24a	Provide registration information for the review, including register name and registration number, or state that the review was not registered.	2
	24b	Indicate where the review protocol can be accessed, or state that a protocol was not prepared.	2
	24c	Describe and explain any amendments to information provided at registration or in the protocol.	2
Support	25	Describe sources of financial or non-financial support for the review, and the role of the funders or sponsors in the review.	12
Competing interests	26	Declare any competing interests of review authors.	12
Availability of data, code and other materials	27	Report which of the following are publicly available and where they can be found: template data collection forms; data extracted from included studies; data used for all analyses; analytic code; any other materials used in the review.	12

Figure S1. Risk of bias assessment, for each outcome, for RCTs. ROB2 tool. [1-3]

Unique ID	Study ID	Experimental	Comparator	Outcome	Weight	D1	D2	D3	D4	D5	Overall
Gong	Gong et al	Navigation	Conventional	Accuracy MP	1	!	+	+	+	!	!
Zhang	Zhang	Navigation	Conventional	Accuracy MP	1	+	+	+	+	!	!
GongCN	Gong	navigation	conventional	Cheeck Numbness	1	!	+	+	+	!	!
GongH	Gong	Navigation	Conventional	Hospitalization	1	!	+	+	+	!	!
GongOT	Gong	Navigation	Conventional	Operative Time	1	!	+	+	+	!	!
ZhangOT	Zhang	Navigation	conventional	Operative Time	1	+	+	+	+	!	!
ChengOT	Cheng	Navigation	Conventional	Operative Time	1	!	+	+	+	+	!
ZhangMO	Zhang	Navigation	Conventional	Mouth Opening	1	+	+	+	+	!	!
ChengMO	Cheng	navigation	conventional	Mouth Opening	1	!	+	+	+	+	!
GongAoB	Gong	Navigation	Conventional	Amount of bleeding	1	!	+	+	+	!	!
ChengAoB	Cheng	navigation	conventional	Amount of Bleeding	1	!	+	+	+	+	!
GongAD	Gong	navigation	conventional	Average deviation	1	!	+	+	+	!	!
ZhangAD	Zhang	navigation	conventional	Average deviation	1	+	+	+	+	!	!
ChengAD	Cheng	navigation	conventional	Average deviation	1	!	+	+	+	!	!
ZhangIO	Zhang	Navigation	Conventional	Accuracy IO	1	+	+	+	+	!	!

Figure S2. Risk of bias assessment, for each outcome, for retrospective studies. ROBINS-I tool.[4, 5]

Risk of bias domains

Study	D1	D2	D3	D4	D5	D6	D7	Overall
Yang et al. Accuracy IO	-	!	-	+	+	+	-	!
Yang et al. Average Deviation	-	!	-	+	+	+	-	!
Yang et al. Mouth Opening	X	!	-	+	+	+	-	!
Yang et al. Hospital stay	X	!	-	+	+	+	-	!
Yang et al. Cheek numbness	-	!	-	+	+	+	-	!
Yang et al. Operative time	-	!	-	+	+	+	-	!
Bao T. et al. Accuracy MP	+	!	-	+	+	+	-	!
Bao T. et al. Accuracy IO	+	!	-	+	+	+	-	!

Domains:
D1: Bias due to confounding.
D2: Bias due to selection of participants.
D3: Bias in classification of interventions.
D4: Bias due to deviations from intended interventions.
D5: Bias due to missing data.
D6: Bias in measurement of outcomes.
D7: Bias in selection of the reported result.

Judgement
! Critical
X Serious
- Moderate
+ Low

Figure S3. Certainty of evidence for each outcome, GRADE table [1-5]

Intraoperative Surgical Navigation compared to Conventional surgery for treating ZMC fractures

Bibliography:

Certainty assessment							Summary of findings				
							Study event rates (%)			Anticipated absolute effects	
Participants (studies) Follow-up	Risk of bias	Inconsistency	Indirectness	Imprecision	Publication bias	Overall certainty of evidence	With Conventional surgery	With Intraoperative Surgical Navigation	Relative effect (95% CI)	Risk with Conventional surgery	Risk difference with Intraoperative Surgical Navigation
**Zygomatic eminence accuracy (assessed with: mm)**											
123 (3 observational studies)	serious[a]	serious[b]	not serious	serious[c]	none	⊕◯◯◯ Very low	59	64	-		MD **0.39 mm lower** (2.23 lower to 1.44 higher)
**Infraorbital rim accuracy (assessed with: mm)**											
73 (3 observational studies)	serious[a,d]	serious[b]	not serious	serious[c]	none	⊕◯◯◯ Very low	34	39	-		MD **0.66 mm lower** (2.19 lower to 0.87 higher)
**Average deviation of the zygomatic bone (assessed with: mm)**											
164 (4 observational studies)	serious[d]	serious[b]	not serious	serious[c]	none	⊕◯◯◯ Very low	82	82	-		MD **0.64 mm lower** (0.95 lower to 0.32 lower)
**Operative time (assessed with: min)**											
164 (4 observational studies)	serious[d]	not serious	not serious	serious[c]	none	⊕◯◯◯ Very low	82	82	-		MD **3.03 minutes higher** (5.62 lower to 11.67 higher)
**Mouth opening (assessed with: mm)**											
86 (3 observational studies)	serious[d]	not serious	not serious	serious[e]	none	⊕◯◯◯ Very low	43	43	-		MD **0.59 mm higher** (0.36 lower to 1.55 higher)
**Postoperative stay (assessed with: day)**											
106 (2 observational studies)	serious[d]	not serious	not serious	very serious[e]	none	⊕◯◯◯ Very low	53	53	-		MD **0.19 day fewer** (5.31 fewer to 4.94 more)
**Amount of bleeding (assessed with: mL)**											
116 (2 RCTs)	not serious	serious[f]	not serious	very serious[e]	none	⊕◯◯◯ Very low	58	58	-		MD **14.85 mL higher** (53.92 lower to 83.63 higher)
**Cheek numbness**											
106 (2 observational studies)	serious[d]	serious[f]	not serious	very serious[c]	none	⊕◯◯◯ Very low	13/53 (24.5%)	12/53 (22.6%)	**OR 0.91** (0.00 to 2121.97)	25 per 100	**2 fewer per 100** (from -- to 75 more)

CI: confidence interval; **MD:** mean difference; **OR:** odds ratio

Explanations

a. Bao T et al. has high risk of bias
b. High heterogeneity
c. Small sample size
d. Yang et al. has high risk of bias
e. Wide confidence intervals, small sample size.
f. Large variation in effect

Figure S4 Forest plot for the postoperative maximum mouth opening comparing the Intraoperative Surgical Navigation and the Conventional surgery groups (0.5 correlation). MD, mean difference. CI, confidence interval. mm, millimeters. [1, 2, 5]

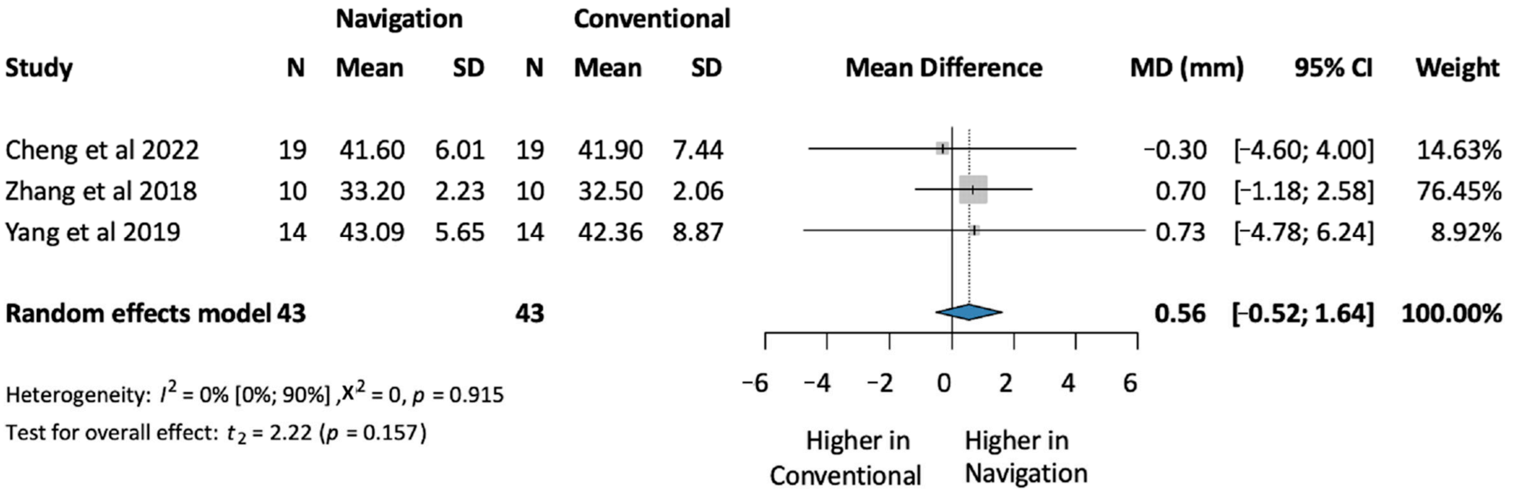

Figure S5 Forest plot for the postoperative maximum mouth opening comparing the Intraoperative Surgical Navigation and the Conventional surgery groups (1 correlation). MD, mean difference. CI, confidence interval. mm, millimeters. [1, 2, 5]

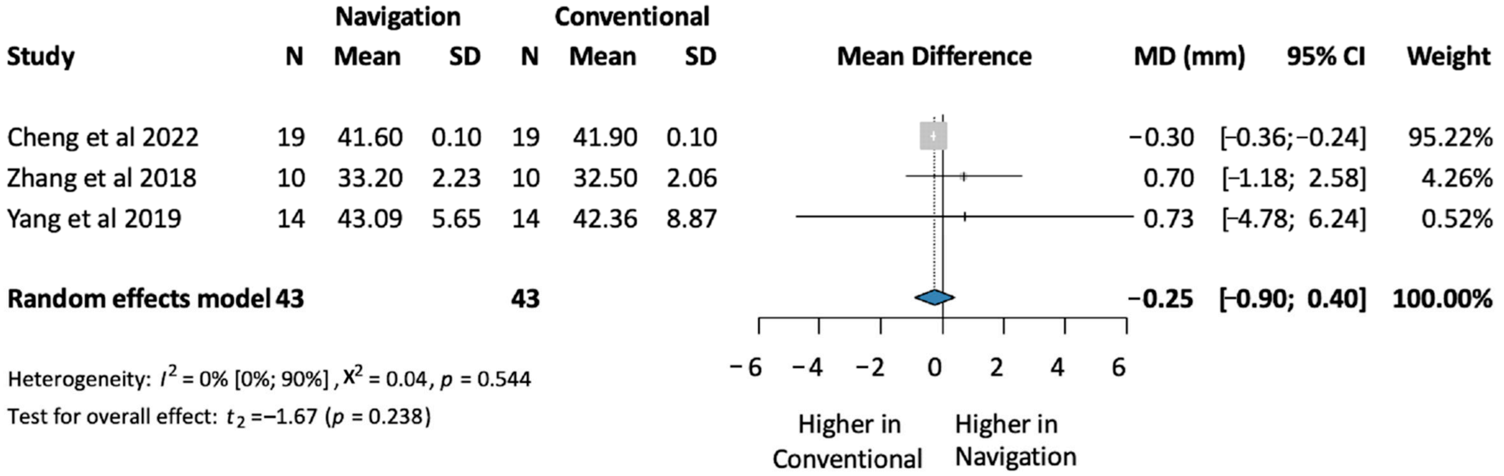

Figure S6 Forest plot for the postoperative maximum mouth opening comparing the Intraoperative Surgical Navigation and the Conventional surgery groups (-1 correlation). MD, mean difference. CI, confidence interval. mm, millimeters. [1, 2, 5]

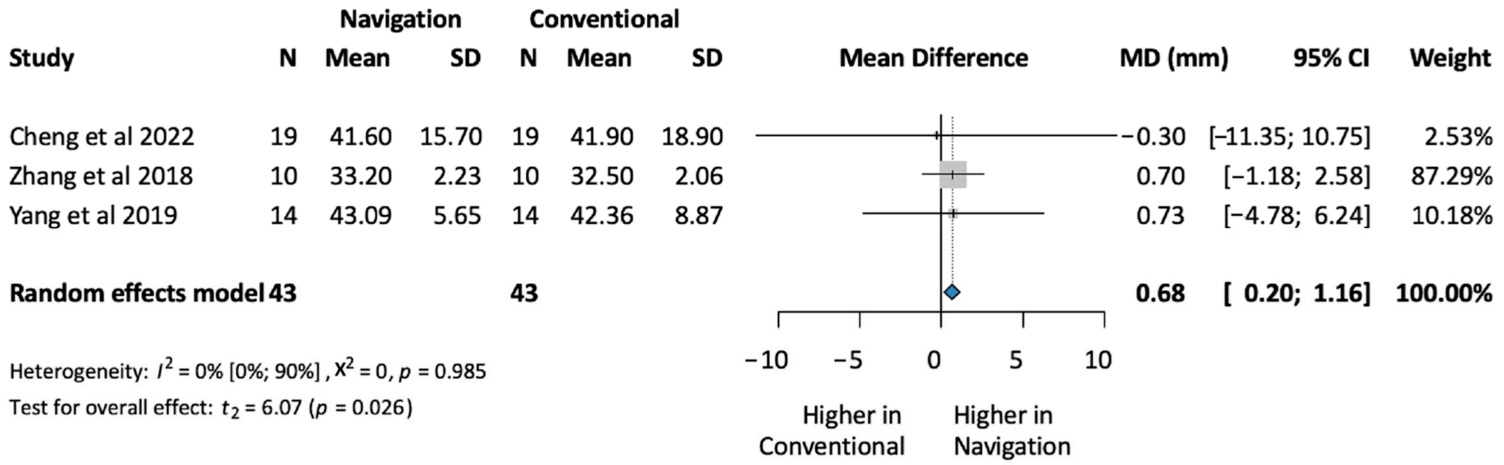

Figure S7 Forest plot for the postoperative maximum mouth opening comparing the Intraoperative Surgical Navigation and the Conventional surgery groups (-0.5 correlation). MD, mean difference. CI, confidence interval. mm, millimeters. [1, 2, 5]

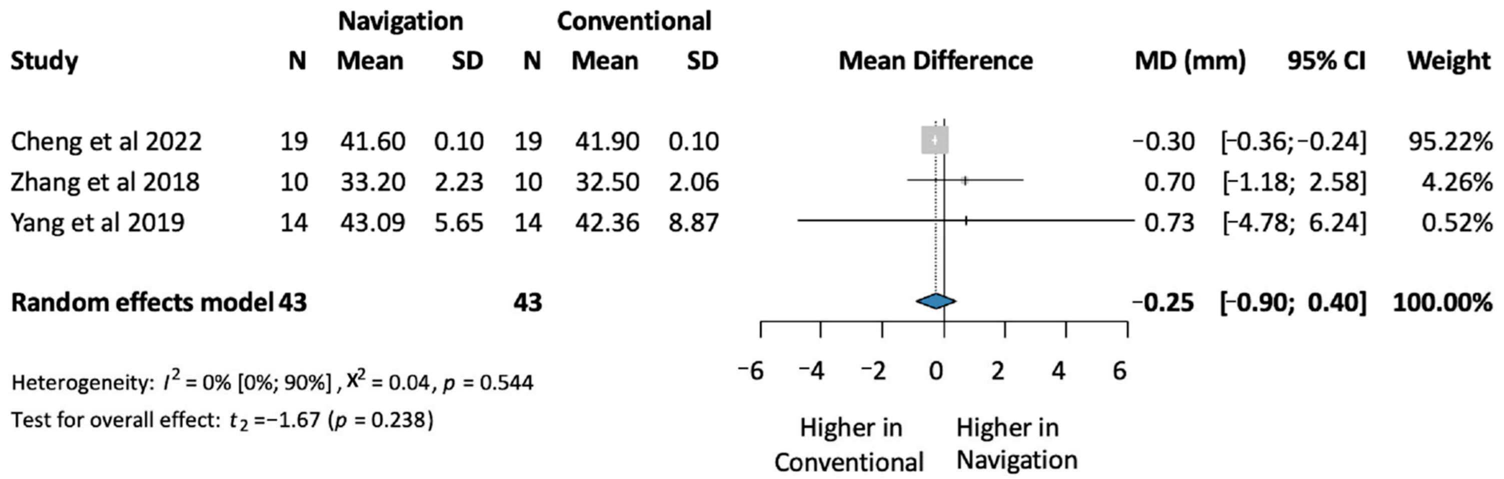

Figure S8. Forest plot for the postoperative stay comparing the Intraoperative Surgical Navigation and the Conventional surgery groups. MD, mean difference (days). CI, confidence interval.[3, 5]

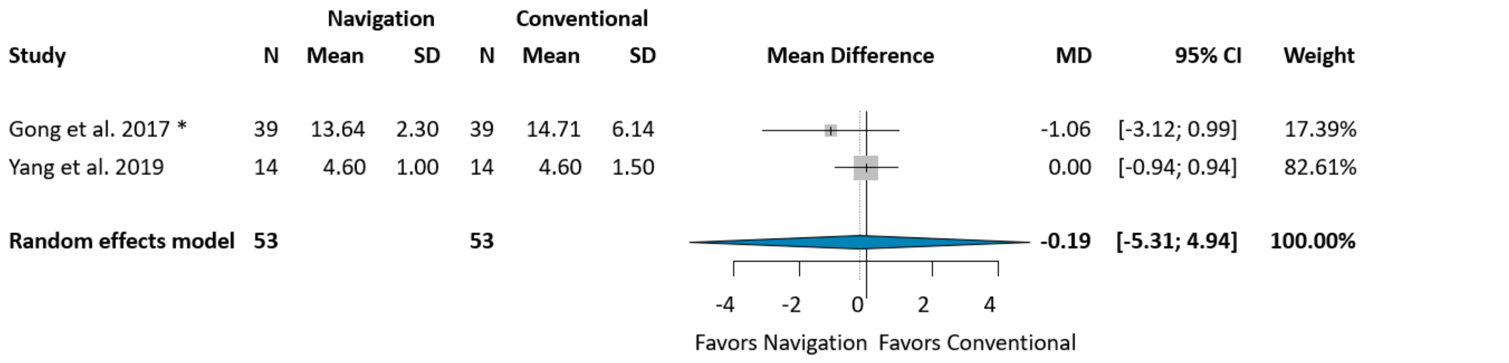

If a study is indicated with * , then its mean and/or standard deviation is estimated from median, quartiles or minimum, maximum values. See raw data and methods.

Figure S9. Forest plot for the amount of bleeding comparing the Intraoperative Surgical Navigation and the Conventional surgery groups. MD, mean difference (milliliters). CI, confidence interval. [2, 3]

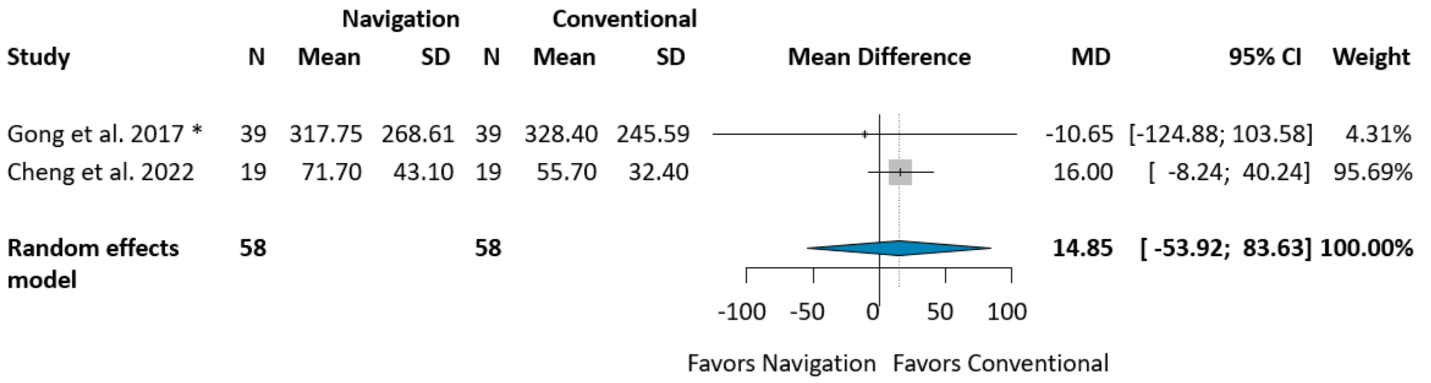

If a study is indicated with * , then its mean and/or standard deviation is estimated from median, quartiles or minimum, maximum values. See raw data and methods.

Figure S10. Forest plot for the cheek numbness comparing the Intraoperative Surgical Navigation and the Conventional surgery groups. OR, odds ratio. CI, confidence interval.[3, 5]

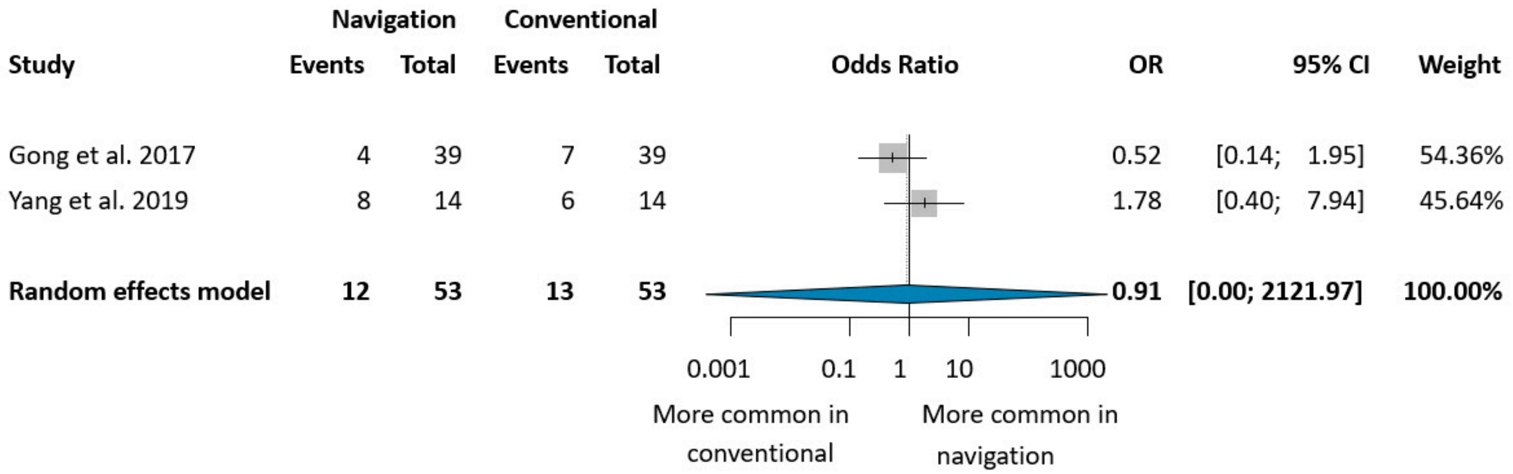

1. Zhang, X., et al., *Surgical navigation improves reductions accuracy of unilateral complicated zygomaticomaxillary complex fractures: a randomized controlled trial.* Sci Rep, 2018. **8**(1): p. 6890.
2. Cheng, M., et al., *Efficacy of surgical navigation in zygomaticomaxillary complex fractures: randomized controlled trial.* Int J Oral Maxillofac Surg, 2022. **51**(9): p. 1180-1187.
3. Gong, X., et al., *Application of a Computer-Assisted Navigation System (CANS) in the Delayed Treatment of Zygomatic Fractures: A Randomized Controlled Trial.* J Oral Maxillofac Surg, 2017. **75**(7): p. 1450-1463.
4. Bao, T., et al., *Quantitative assessment of symmetry recovery in navigation-assisted surgical reduction of zygomaticomaxillary complex fractures.* J Craniomaxillofac Surg, 2019. **47**(2): p. 311-319.
5. Yang, C., et al., *Application of Computer-Assisted Navigation System in Acute Zygomatic Fractures.* Ann Plast Surg, 2019. **82**(1S Suppl 1): p. S53-S58.
